# Weight Function Method for Stress Intensity Factors of Semi-Elliptical Surface Cracks on Functionally Graded Plates Subjected to Non-Uniform Stresses

**DOI:** 10.3390/ma13143155

**Published:** 2020-07-15

**Authors:** Kun-Pang Kou, Jin-Long Cao, Yang Yang, Chi-Chiu Lam

**Affiliations:** Department of Civil and Environmental Engineering, Faculty of Science and Technology, University of Macau, Macau 999078, China; kpkou@um.edu.mo (K.-P.K.); yangyang.liju@hotmail.com (Y.Y.); fstccl@um.edu.mo (C.-C.L.)

**Keywords:** functionally graded plates, weight function method, stress intensity factors, non-uniform stress distributions, semi-elliptical surface cracks, finite element analysis

## Abstract

In this paper, a weight function method based on the first four terms of a Taylor’s series expansion is proposed to determine the stress intensity factors of functionally graded plates with semi-elliptical surface cracks. Cracked surfaces that are subjected to constant, linear, parabolic and cubic stress fields are considered. The weight functions for the surface, deepest and general points on the crack faces of long and deep cracked functionally graded plates are derived, which has never been done before in the literature. The accuracy of the method in this study is then validated by comparing the results with those of finite element modeling. The numerical results indicate that the derived weight functions are highly accurate and robust enough to predict the stress intensity factors for cracked functionally graded plates subjected to non-uniform stress distributions. The weight function method is therefore a time-saving technique and suitable for handling non-uniform stress fields.

## 1. Introduction

Functionally graded material (FGM) is a new type of composite material, which consists of two or more types of material with different properties. The use of advanced material compounding technology to build the composition and structure of the intermediate shows continuous changes in gradients, without an obvious interface at the intermediate layer, with gradient changes that appear in the properties and functions of materials along the thickness direction [1]. The superior physical, mechanical and thermal properties of FGMs mean that they have been rapidly developed and widely applied in the aerospace, machinery, weapons, medical, electronics and other industries in the past few decades. Research on the behaviors of FGMs has been also ongoing since the concept of FGM was first proposed by Yamanouchi et al. [2].

The manufacturing process of functionally graded (FG) members may result in different shaped cracks on the surface of the slab due to thermal stress or the improper use of reheating and rolling parameters, which leads to mechanical deformation. Since the shapes of the cracks on FG structures are generally irregular and cannot be directly simulated, the irregular-shaped cracks are simplified in accordance with BS 7910 [3] as semi-elliptical cracks. The stress intensity factor (SIF) is considered to be a significant parameter for evaluating the safety and predicting the fatigue life of cracked structures or components. While there are many effective ways to calculate SIFs, the finite element method (FEM) is the most prevalent one among the current numerical methods. Walters et al. [4] provided widely accepted solutions of SIFs for semi-elliptical surface cracks on FG plates. Nevertheless, finite element (FE) modeling and mesh generation are extremely time-consuming processes when various stress distributions are taken into account, and it is difficult to calculate the SIFs of all the points on the crack front. To address this shortcoming, an effective analytical technique, namely the weight function method, which is independent of the stress fields, was introduced to determine the SIFs for various loading conditions. The SIFs calculated by the weight function method vary continuously along the crack front. The maximum SIF can be more easily predicted by using this method if the variation of the SIFs along the crack front is minimal and stable. The weight function method was first described by Rice [5]. The algebraic expressions of the weight functions for a homogeneous elastic material were derived by Shen and Glinka [6]. The derived weight functions are related to the geometric characteristics of the cracked members, but independent of the applied stress distributions [7,8], and the accuracy is not affected by crack dimensions, geometries and stress distributions [9]. Therefore, the weight function method is more simple, convenient and efficient in calculating SIFs for a variety of stress distributions compared to the FEM [10]. Moreover, if the stress distribution cannot be denoted by polynomials in practical applications, this effective analytical integration method can be used in lieu [11]. However, the complex mechanical properties of FGMs mean that the weight functions for surface cracks on FG plates have been rarely presented in the literature, especially for the general points on the crack front, which have never been reported in any study.

The maximum SIF has great significance for fracture analyses and fatigue life predictions of cracked FG plates [12]. Thus, the location of the maximum SIF should be focused. In general, the maximum SIF of a homogeneous plate with a semi-elliptical crack usually occurs at the surface point or the deepest point. Nevertheless, owing to the inhomogeneity of FG plates and the changes in applied loads, the maximum SIF of an FG plate with a semi-elliptical crack may be found at any point along the crack front. Therefore, the weight functions for the deepest, surface and general points of a surface crack on an FG plate need to be derived.

In this study, the general expressions of weight functions for an FG plate with a semi-elliptical surface crack are derived in accordance with an expression that uses the first four terms of a Taylor’s series expansion. The weight functions in this work, derived by combining the solutions of constant and linear stress distributions, have a wide range of applications. The newly derived weight functions of the surface, deepest and general points within the range of 0.2 ≤ *a*/*c* ≤ 1.0 and 0.1 ≤ *a*/*t* ≤ 0.8 are validated by comparing the analytical solutions in this study with the FE results for parabolic or cubic stress distributions, where *a*/*c* denotes the aspect ratio and *a*/*t* represents the crack depth ratio. The existing numerical results show that the crack length *c*, crack depth *a* and crack-tip location have a significant impact on the analysis results of crack problems [13,14]; therefore, a wide range of *a*/*c* (0.2, 0.4, 0.6, 0.8, 1.0) and *a*/*t* (0.1, 0.2, 0.3, 0.4, 0.5, 0.6, 0.7, 0.8) are assigned in FE models. According to reference [15], the condition of *h*/*c* = *w*/*c* ≥ 4 and *t* equal to a constant is defined in FE simulations, where *h*, *t* and *w* denote the half height, thickness and half width of the FG plate, respectively. It should be noted that the calculation results (such as the SIFs) have been expressed in non-dimensional ratios for specific cases [16]. The annotation of parameters or symbols used in this study can be found in the “Nomenclature” section of the Supplementary Materials.

## 2. Fracture Analysis of Surface Crack on FG Plates

### 2.1. FE Modelling of Cracked FG Plates

**Figure 1:**
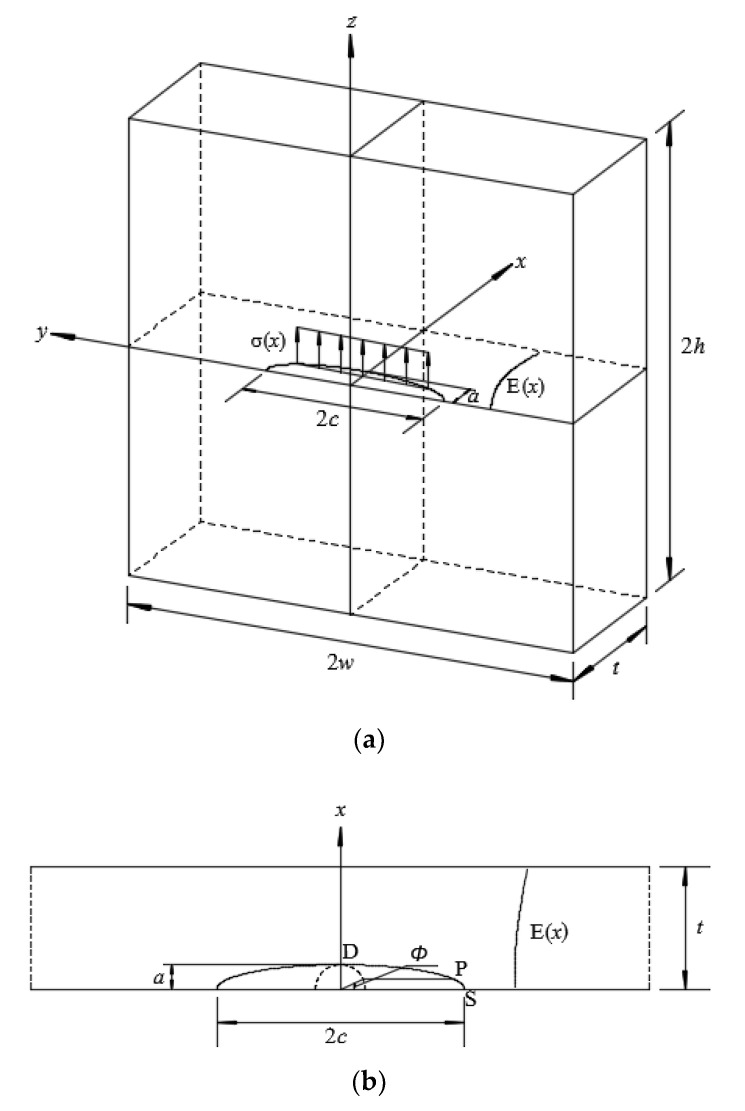
(**a**) Geometry and coordinate system of a functionally graded (FG) plate with a semi-elliptical surface crack subjected to local stress field; (**b**) semi-elliptical surface crack.

Figure 1a shows a three-dimensional FE model for an FG plate with a semi-elliptical surface crack subjected to a local stress field, where *a* and *c* denote the crack depth and half crack length of a semi-elliptical surface crack, respectively, and *σ*(*x*) denotes a local stress distribution perpendicular to the crack face. A detailed semi-elliptical crack face is illustrated in Figure 1b, where points S, P and D represent the surface, general and deepest points on the crack front, respectively, and *ϕ* denotes the parametric angle of the elliptical surface crack. Due to the double symmetry of the model, only one quarter of the cracked plate was numerically modelled [17]. For homogeneous linear elastic material, material properties are defined, such as the Young’s modulus *E* = 210 GPa and Poisson’s ratio *v* = 0.3. For FGMs, the Young’s modulus *E*(*x*) varies continuously along the *x*-coordinates and is governed by an exponential function, as expressed in Equation (1), where *E*_0_ and *E*_1_ denote the Young’s modulus of starting and ending constituents of an FG plate, respectively [18]. Yang et al. [19] proposed that the Young’s modulus of the ceramic-steel FG layer can be described by Equation (1). An FGM for which the Young’s modulus can be described by Equation (1) was used in Li et al.’s study [20], where the FGM was made of ethylene carbon monoxide copolymer.

(1) $$E\left(x\right)=E_{0}\exp\left[\frac{1}{t}\ln\left(\frac{E_{1}}{E_{0}}\right)x\right]$$

Table 1 presents the typical values of aspect ratio (*a*/*c*) and crack depth ratio (*a*/*t*) for the plates with semi-elliptical surface cracks. An FORTRAN program for mesh generation was developed to generate all necessary FE models [21].

**Figure 2:**
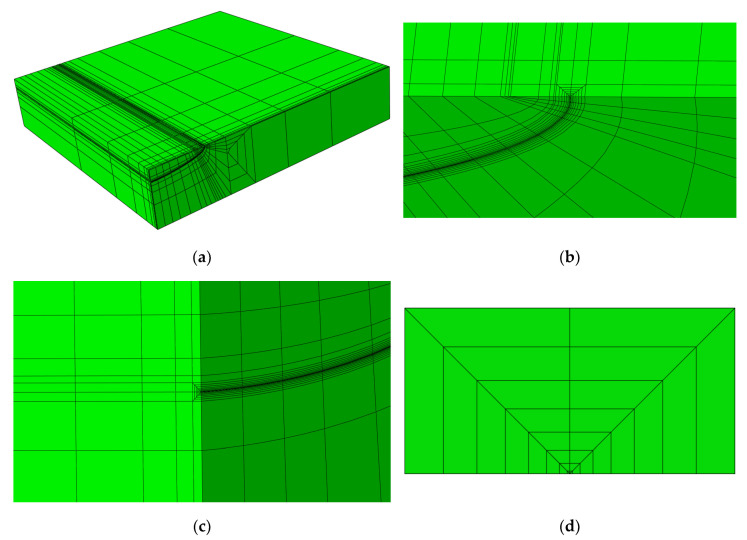
Typical FE mesh of FG plate with semi-elliptical surface crack, *a*/*c* = 0.2, *a*/*t* = 0.2. (**a**) FE mesh of the entire cracked FG plate; (**b**) FE mesh near the surface point; (**c**) FE mesh near the deepest point; (**d**) FE mesh of the near-tip region.

Figure 2 demonstrates a typical mesh of an FE model of an FG plate with a semi-elliptical surface crack; this FE model has 2517 elements and 12,244 nodes. The ratio of *w*/*c* decreases with the increase in *c*; to maintain *w*/*c* ≥ 4, additional elements are added to eliminate the effect of *w*—details are available in the literature [15]; therefore, the number of elements in each FE model is within the range of 2517~2943, and the number of nodes is within the range of 12,244~14,132. In the FE models, the elements of the crack-tip region used were 15-node quadratic triangular prisms (C3D15), all the other elements used were 20-node quadratic bricks (C3D20).

The J-integral method, which is an effective energy-based method, was used to determine the SIFs. In case of linear elasticity, the J-integral is equal to the energy release rate; therefore, the SIF, *K*, can be expressed by using the following equation. All the points on the crack front other than the surface point were treated as plane stress conditions.

(2) $$K=\sqrt{J{E^{\prime }}_{\mathrm{tip}}}$$

where ${E^{\prime }}_{\mathrm{tip}}$ denotes the modified crack-tip Young’s modulus, $E_{\mathrm{tip}}$ denotes the crack-tip Young’s modulus, ${E^{\prime }}_{\mathrm{tip}}=E_{\mathrm{tip}}$ for plane stress condition, and ${E^{\prime }}_{\mathrm{tip}}=E_{\mathrm{tip}}/(1-v^{2})$ for plane strain condition.

SIFs were normalized as follows:

(3) $$F=\frac{K}{\sigma_{0}\sqrt{\pi a/Q}}$$

where *F* is a boundary correction factor (BCF), $\sigma_{0}$ is a nominal stress, and *Q* is the shape factor and denotes the square of the complete elliptic integral of the second kind [11]. The equation of *Q* which varies with the aspect ratio (*a*/*c*), is expressed as follows:

(4) $$Q=1.0+1.464{\left(a/c\right)}^{1.65}$$

Four types of stress distributions are defined that act on crack surfaces:

(5) $$\sigma \left(x\right)=\sigma_{0}{\left(1-\frac{x}{a}\right)}^{n}$$

with n = 0, 1, 2, 3. For n = 0, σ(x) is a constant stress distribution; n = 1, σ(x) is a linear stress distribution; n = 2, σ(x) is a parabolic stress distribution; and n = 3, σ(x) is a cubic stress distribution.

### 2.2. Validation of FE Models

In order to validate the effect of the present FE models to simulate cases under non-uniform stress fields, linear and parabolic stress fields are assumed to act on the crack face. The results of the FE models for the deepest and surface points were validated against the existing data [11]. The calculated BCFs are found to be in good agreement with the results in reference [11], in which the largest difference is still less than 2.61% and most are less than 2%, as shown in Table 2.

**Figure 3:**
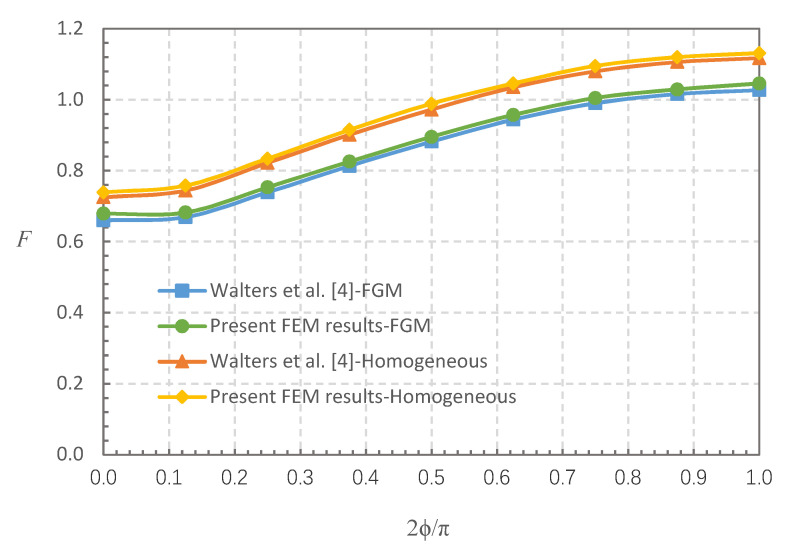
Comparison of BCFs (*F*): finite element method (FEM) results in this study vs. results in reference [4], for *a*/*t* = 0.2, *a*/*c* = 1/3, *E*(1)/*E*(0) = 0.2, and homogeneous material.

**Figure 4:**
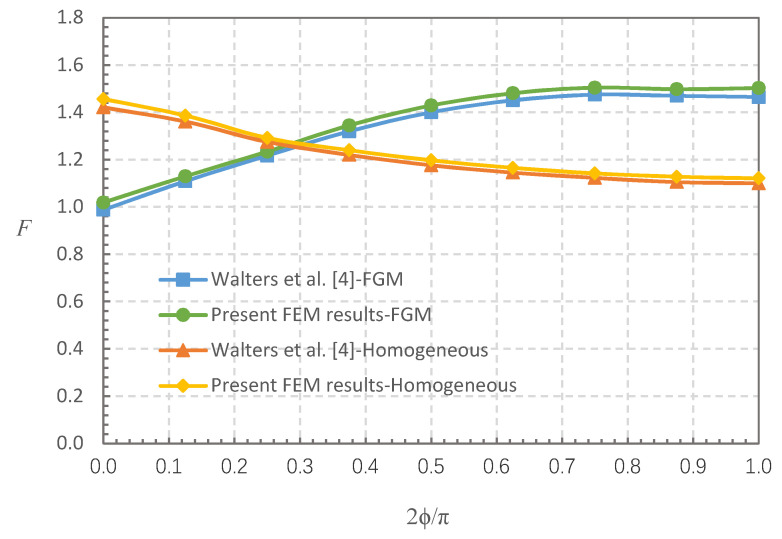
Comparison of BCFs (*F*): FEM results in this study vs. results in reference [4], for *a*/*t* = 0.8, *a*/*c* = 1, *E*(1)/*E*(0) = 5, and homogeneous material.

This section discusses FGMs for which the elastic modulus conforms to Equation (1), and cracked FG plates that are subjected to a uniform tensile stress field. Two cases were studied: (i) *a*/*t* = 0.2, *a*/*c* = 1/3, *E*(1)/*E*(0) = 0.2, and (ii) *a*/*t* = 0.8, *a*/*c* = 1, *E*(1)/*E*(0) = 5. The results of the FGM and corresponding homogeneous material cases were compared with the findings in reference [4]. Figure 3 and Figure 4 show that the FEM results for the FGMs and homogeneous material are in good agreement with the FEM results in reference [4], in which the maximum difference is within 3.13% and most are within 2%.

The results show that the generated FE models are suitable for addressing cases with non-uniform stress fields and calculating the SIFs of surface cracks found on FG plates.

## 3. Weight Functions for Calculating SIFs of Cracked FG Plates

### 3.1. General Weight Function Forms

Although there are many numerical methods that can be used to accurately calculate SIFs, they can only calculate one stress distribution and one crack length at a time; therefore, the process is time-consuming. Since the weight function method is independent of the stress distributions, it can simplify the calculation process of SIFs with various stress distributions. SIFs were calculated by using the integration of the weight function m(x,a) multiplied by the stress distribution σ(x), and the integration was performed over the crack length *a*.

(6) $$K=\int_{0}^{a}m\left(x,a\right)\sigma \left(x\right)\;dx$$

The SIFs for a given cracked body under any stress distribution can be obtained after the weight function is determined due to the uniqueness of the weight function [22].

The relationship between the crack opening displacement u(x,a) and the weight function m(x,a) was derived by Rice [5], it is expressed as follows:

(7) $$m\left(x,a\right)=\frac{E_{\mathrm{tip}}}{K_{r}(a)}\frac{\partial u\left(x,a\right)}{\partial a}$$

where $K_{r}(a)$ denotes the reference SIF that related to the crack length *a*.

Even if the Young’s modulus of an FGM varies with the *x*-coordinates, *E* = *E*(*x*), the near-tip field of the FGM must be identical to a homogeneous material, which means that the Young’s modulus at the crack tip of an FGM is equal to a constant $E_{\mathrm{tip}}$ [7].

The four-term weight function is derived from reference [7]:

(8) $$m\left(x,a\right)=\sqrt{\frac{2}{\pi a}}\left[{\left(1-\frac{x}{a}\right)}^{-1/2}+D_{1}{\left(1-\frac{x}{a}\right)}^{1/2}+D_{2}{\left(1-\frac{x}{a}\right)}^{3/2}+D_{3}{\left(1-\frac{x}{a}\right)}^{5/2}\right]$$

where $D_{1}$, $D_{2}$ and $D_{3}$ are the coefficients of the derived weight function.

Equation (6) is transformed into the following form by introducing Equations (5) and (8) into (6).

(9) $$K=\sqrt{\frac{2a}{\pi }}\sigma_{0}\left(\frac{2}{2n+1}+\frac{2}{2n+3}D_{1}+\frac{2}{2n+5}D_{2}+\frac{2}{2n+7}D_{3}\right)$$

The weight function for the deepest point is expressed by using the following equation:

(10) $$m_{D}\left(x,a\right)=\sqrt{\frac{2}{\pi a}}\left[{\left(1-\frac{x}{a}\right)}^{-1/2}+D_{D1}{\left(1-\frac{x}{a}\right)}^{1/2}+D_{D2}{\left(1-\frac{x}{a}\right)}^{3/2}+D_{D3}{\left(1-\frac{x}{a}\right)}^{5/2}\right]$$

where $D_{D1}$, $D_{D2}$ and $D_{D3}$ are the weight function coefficients for the deepest point.

The weight function for the surface point is expressed as follows, according to reference [6].

(11) $$m_{S}\left(x,a\right)=\frac{2}{\sqrt{\pi x}}\left[{\left(\frac{x}{a}\right)}^{-1/2}+D_{S1}{\left(\frac{x}{a}\right)}^{1/2}+D_{S2}{\left(\frac{x}{a}\right)}^{3/2}+D_{S3}{\left(\frac{x}{a}\right)}^{5/2}\right]$$

where $D_{S1}$, $D_{S2}$ and $D_{S3}$ are the weight function coefficients for the surface point.

In this study, since the stress distributions on the crack face and the elastic modulus along the crack front are variable, the maximum SIF may occur at any point on the crack front. Therefore, the weight function for the general point is required. However, the weight function for the general point on a semi-elliptical surface crack of an FG plate has not been reported in the literature. Therefore, closed-form local weight functions for the general point P within the range of 0°<ϕ<90° are newly derived from reference [23].

The weight functions for the general point P are expressed as follows:

For 0≤x≤asinϕ:

(12) $$m_{P1}\left(x,a\right)=\sqrt{\frac{2}{\pi a\sin\phi }}\left[{\left(1-\frac{x}{a\sin\phi }\right)}^{-\frac{1}{2}}+D_{P1}{\left(1-\frac{x}{a\sin\phi }\right)}^{\frac{1}{2}}+D_{P2}{\left(1-\frac{x}{a\sin\phi }\right)}^{\frac{3}{2}}\right]$$

For asinϕ≤x≤a:

(13) $$m_{P2}\left(x,a\right)=\sqrt{\frac{2}{\pi a\sin\phi }}\left[{\left(\frac{x}{a\sin\phi }-1\right)}^{-\frac{1}{2}}+D_{P3}{\left(\frac{x}{a\sin\phi }-1\right)}^{\frac{1}{2}}+D_{P4}{\left(\frac{x}{a\sin\phi }-1\right)}^{\frac{3}{2}}\right]$$

where $D_{P1}$, $D_{P2}$, $D_{P3}$ and $D_{P4}$ are the weight function coefficients for the general point.

Since the present FE simulations and derivation of weight functions are based on the condition of *h*/*c* = *w*/*c* ≥ 4, that is, the crack size is small enough compared to the FG plate [15]. Therefore, for a surface crack with depth *a*, it can be proved (see Supplementary Materials) that the curvature of the crack contour at the surface (*x* = 0) vanishes [24]:

(14) $$\frac{\partial^{2}u(x,a)}{\partial x^{2}}|{}_{x=0}=0$$

The second derivative of the weight function for the deepest point is therefore equal to zero at *x* = 0 according to Equations (7) and (14), as follows. The detailed derivation is shown in the Supplementary Materials.

(15) $$\frac{\partial^{2}m_{D}(x,a)}{\partial x^{2}}|{}_{x=0}=0$$

The additional condition for the deepest point is obtained from Equation (15), as follows:

(16) $$D_{D1}-3D_{D2}-15D_{D3}=3$$

Due to the weight function for the surface point of a semi-elliptical surface crack is derived from the weight function for the embedded penny-shape crack; therefore, the weight function in Equation (11) must vanish at *x* = *a* [25], as follows; details are available in the Supplementary Materials.

(17) $$m_{S}(x,a)|{}_{x=a}=0$$

The additional condition for the surface point is obtained from Equation (17), as follows:

(18) $$D_{S1}+D_{S2}+D_{S3}=-1$$

The deepest point (ϕ=π/2) and surface point (ϕ = 0) are special cases of the general points. The additional conditions for the general point are obtained by analogy with Equations (15) and (17) [26]. The detailed explanation is shown in Supplementary Materials.

(19) $$m_{P2}(x,a)|{}_{x=a}=0$$

(20) $$\frac{\partial^{2}m_{P1}(x,a)}{\partial x^{2}}|{}_{x=0}=0$$

The additional conditions for the general point are given:

(21) $$-D_{P1}+3D_{P2}=-3$$

(22) $$1+D_{P3}\left(\frac{1}{\sin\phi }-1\right)+D_{P4}{\left(\frac{1}{\sin\phi }-1\right)}^{2}=0$$

### 3.2. Weight Function for Deepest Point of Surface Crack on FG Plate

The reference SIFs for constant stress distribution are determined by using:

(23) $$K_{r1}^{D}=\sigma_{0}\sqrt{\frac{\pi a}{Q}}Y_{0}$$

The reference SIFs for linear stress distribution are determined with:

(24) $$K_{r2}^{D}=\sigma_{0}\sqrt{\frac{\pi a}{Q}}Y_{1}$$

The coefficient equations of the weight function for the deepest point are obtained by substituting Equations (23) and (24) into (9) and adding Equation (16):

(25) $$D_{D1}-3D_{D2}-15D_{D3}=3$$

(26) $$\frac{2}{3}D_{D1}+\frac{2}{5}D_{D2}+\frac{2}{7}D_{D3}=\frac{\pi }{\sqrt{2Q}}Y_{0}-2$$

(27) $$\frac{2}{5}D_{D1}+\frac{2}{7}D_{D2}+\frac{2}{9}D_{D3}=\frac{\pi }{\sqrt{2Q}}Y_{1}-\frac{2}{3}$$

The coefficients $D_{D1}$, $D_{D2}$ and $D_{D3}$ are determined by solving Equations (25)–(27). The weight function ($m_{D}(x,a)$) for the deepest point can be obtained by substituting Equations (28)–(30) into Equation (10).

(28) $$D_{D1}=\frac{\pi }{\sqrt{2Q}}\left(10.389Y_{0}-14.766Y_{1}\right)-10.999$$

(29) $$D_{D2}=\frac{\pi }{\sqrt{2Q}}\left(-17.861Y_{0}+29.521Y_{1}\right)+16.332$$

(30) $$D_{D3}=\frac{\pi }{\sqrt{2Q}}\left(4.265Y_{0}-6.889Y_{1}\right)-4.200$$

### 3.3. Weight Function for Surface Point of Surface Crack on FG Plate

The reference SIFs for the constant stress distribution can be determined by using:

(31) $$K_{r1}^{S}=\sigma_{0}\sqrt{\frac{\pi a}{Q}}F_{0}$$

The reference SIFs for linear stress distribution can be determined with:

(32) $$K_{r2}^{S}=\sigma_{0}\sqrt{\frac{\pi a}{Q}}F_{1}$$

The coefficient equations of the weight function for the surface point are obtained by substituting Equations (31) and (32) into Equation (9) and adding Equation (18):

(33) $$D_{S1}+D_{S2}+D_{S3}=-1$$

(34) $$\frac{2}{3}D_{S1}+\frac{2}{5}D_{S2}+\frac{2}{7}D_{S3}=\frac{\pi }{\sqrt{2Q}}F_{0}-2$$

(35) $$\frac{2}{5}D_{S1}+\frac{2}{7}D_{S2}+\frac{2}{9}D_{S3}=\frac{\pi }{\sqrt{2Q}}F_{1}-\frac{2}{3}$$

The coefficients $D_{S1}$, $D_{S2}$ and $D_{S3}$ are determined by solving Equations (33)–(35). The weight function ($m_{S}(x,a)$) for the surface point can be obtained by substituting Equations (36)–(38) into Equation (11).

(36) $$D_{S1}=\frac{\pi }{\sqrt{2Q}}\left(16.411F_{0}-29.531F_{1}\right)-15.003$$

(37) $$D_{S2}=\frac{\pi }{\sqrt{2Q}}\left(-45.945F_{0}+98.461F_{1}\right)+35.008$$

(38) $$D_{S3}=\frac{\pi }{\sqrt{2Q}}(29.537F_{0}-68.919F_{1})-21.004$$

### 3.4. Weight Function for General Point of Surface Crack on FG Plate

The reference SIFs for constant stress distribution can be determined with:

(39) $$K_{r1}^{P}=\sigma_{0}\sqrt{\frac{\pi a}{Q}}Z_{0}$$

The reference SIFs for linear stress distribution can be determined by using:

(40) $$K_{r2}^{P}=\sigma_{0}\sqrt{\frac{\pi a}{Q}}Z_{1}$$

The coefficient equations of the weight function for the general point are obtained by substituting Equations (5), (12), (13), (39) and (40) into Equation (6) and adding Equations (21) and (22). Detailed derivation process is available in the Supplementary Materials.

(41) $$3-D_{P1}+3D_{P2}=0$$

(42) $$1+D_{P3}\left(\frac{1}{\sin\phi }-1\right)+D_{P4}{\left(\frac{1}{\sin\phi }-1\right)}^{2}=0$$

(43) $$\left[1+\frac{1}{3}D_{P1}+\frac{1}{5}D_{P2}\right]+[{\left(\frac{1}{\sin\phi }-1\right)}^{\frac{1}{2}}+\frac{1}{3}D_{P3}{\left(\frac{1}{\sin\phi }-1\right)}^{\frac{3}{2}}+\frac{1}{5}D_{P4}{\left(\frac{1}{\sin\phi }-1\right)}^{\frac{5}{2}}]=\pi \sqrt{\frac{1}{8Q\sin\phi }}Z_{0}$$

(44) $$\begin{array}{l} \left[1+\frac{1}{3}D_{P1}+\frac{1}{5}D_{P2}\right]-\left[\frac{2}{3}\sin\phi +\frac{2}{15}\sin\phi D_{P1}+\frac{2}{35}\sin\phi D_{P2}\right]+[\frac{2}{3}\sin\phi {\left(\frac{1}{\sin\phi }-1\right)}^{\frac{3}{2}}\\ \;+\frac{2}{15}\sin\phi D_{P3}{\left(\frac{1}{\sin\phi }-1\right)}^{\frac{5}{2}}+\frac{2}{35}\sin\phi D_{P4}{\left(\frac{1}{\sin\phi }-1\right)}^{\frac{7}{2}}]=\pi \sqrt{\frac{1}{8Q\sin\phi }}Z_{1} \end{array}$$

The coefficients $D_{P1}$, $D_{P2}$, $D_{P3}$ and $D_{P4}$ are determined by solving Equations (41)–(44). The weight functions ($m_{P1}(x,a)$ and $m_{P2}(x,a)$) for the general point can be determined by substituting Equations (45)–(48) into Equations (12) and (13).

(45) $$\begin{array}{l} D_{P1}=[32f^{5}\sin\phi -128f^{\frac{7}{2}}\sin\phi -96f^{\frac{9}{2}}\sin\phi +168f^{\frac{7}{2}}-210f^{\frac{7}{2}}V_{1}{\left(\frac{1}{Q\sin\phi }\right)}^{\frac{1}{2}}\\ \;+120f^{\frac{9}{2}}V_{0}\sin\phi {\left(\frac{1}{Q\sin\phi }\right)}^{\frac{1}{2}}]/\left[2f\left(16f^{\frac{5}{2}}\sin\phi +24f^{\frac{7}{2}}\sin\phi -42f^{\frac{5}{2}}\right)\right] \end{array}$$

(46) $$\begin{array}{l} D_{P2}=[32f^{5}\sin\phi -224f^{\frac{7}{2}}\sin\phi -240f^{\frac{9}{2}}\sin\phi +420f^{\frac{7}{2}}-210f^{\frac{7}{2}}V_{1}{\left(\frac{1}{Q\sin\phi }\right)}^{\frac{1}{2}}\\ \;+120f^{\frac{9}{2}}V_{0}\sin\phi {\left(\frac{1}{Q\sin\phi }\right)}^{\frac{1}{2}}]/\left[6f\left(16f^{\frac{5}{2}}\sin\phi +24f^{\frac{7}{2}}\sin\phi -42f^{\frac{5}{2}}\right)\right] \end{array}$$

(47) $$\begin{array}{l} D_{P3}=[96f^{2}\sin\phi -96f^{\frac{5}{2}}\sin\phi -192f^{\frac{7}{2}}\sin\phi +252f^{\frac{5}{2}}-315f^{2}V_{0}{\left(\frac{1}{Q\sin\phi }\right)}^{\frac{1}{2}}+315f^{2}V_{1}{\left(\frac{1}{Q\sin\phi }\right)}^{\frac{1}{2}}\\ \;+120f^{2}V_{0}\sin\phi {\left(\frac{1}{Q\sin\phi }\right)}^{\frac{1}{2}}]/\left[f\left(16f^{\frac{5}{2}}\sin\phi +24f^{\frac{7}{2}}\sin\phi -42f^{\frac{5}{2}}\right)\right] \end{array}$$

(48) $$\begin{array}{l} D_{P4}=[-96f\sin\phi +80f^{\frac{3}{2}}\sin\phi +168f^{\frac{5}{2}}\sin\phi -210f^{\frac{3}{2}}+315fV_{0}{\left(\frac{1}{Q\sin\phi }\right)}^{\frac{1}{2}}-315fV_{1}{\left(\frac{1}{Q\sin\phi }\right)}^{\frac{1}{2}}\\ \;-120f\sin\phi V_{0}{\left(\frac{1}{Q\sin\phi }\right)}^{\frac{1}{2}}]/\left[f\left(16f^{\frac{5}{2}}\sin\phi +24f^{\frac{7}{2}}\sin\phi -42f^{\frac{5}{2}}\right)\right] \end{array}$$

where $f=\frac{1}{\sin\phi }-1$, $V_{0}=\frac{\pi }{2\sqrt{2}}Z_{0}$, $V_{1}=\frac{\pi }{2\sqrt{2}}Z_{1}$

### 3.5. Validation of Derived Weight Function

**Figure 5:**
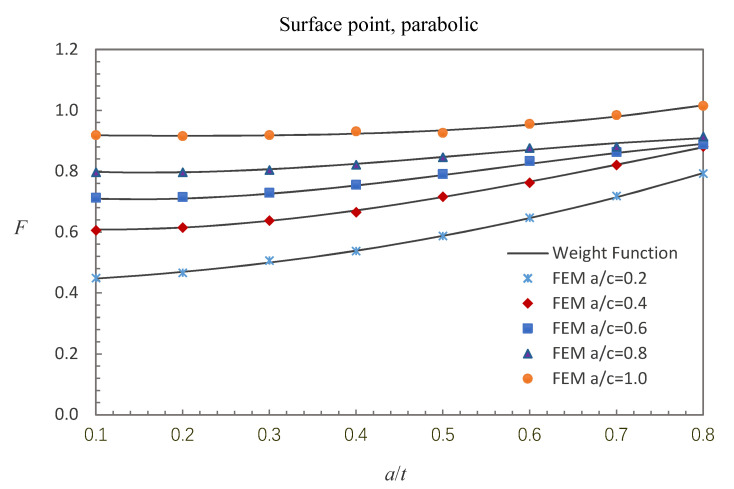
Comparison of weight function and FE results in this study with parabolic stress distribution (surface point).

**Figure 6:**
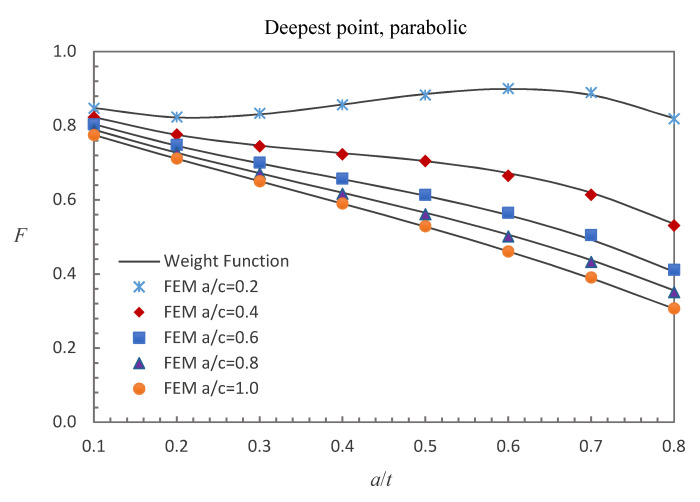
Comparison of weight function and FE results in this study with parabolic stress distribution (deepest point).

**Figure 7:**
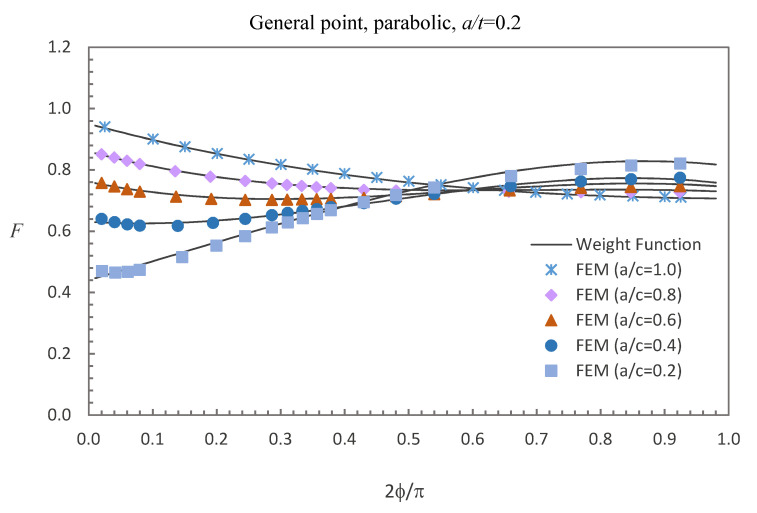
Comparison of weight function and FE results in this study with parabolic stress distribution (general point).

**Figure 8:**
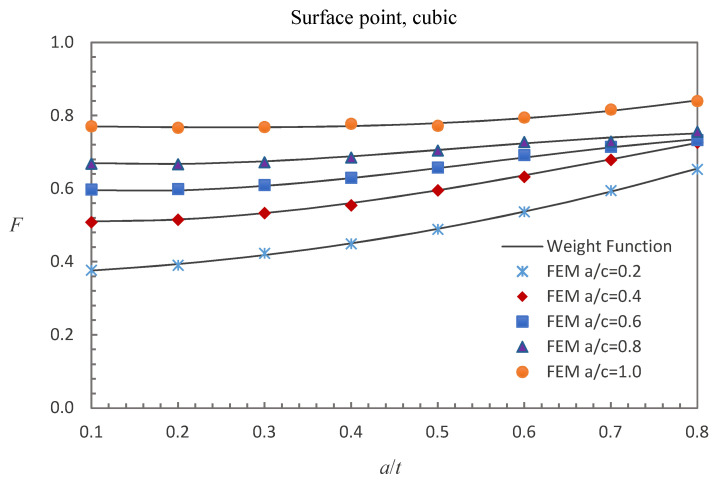
Comparison of weight function and FE results in this study with cubic stress distribution (surface point).

**Figure 9:**
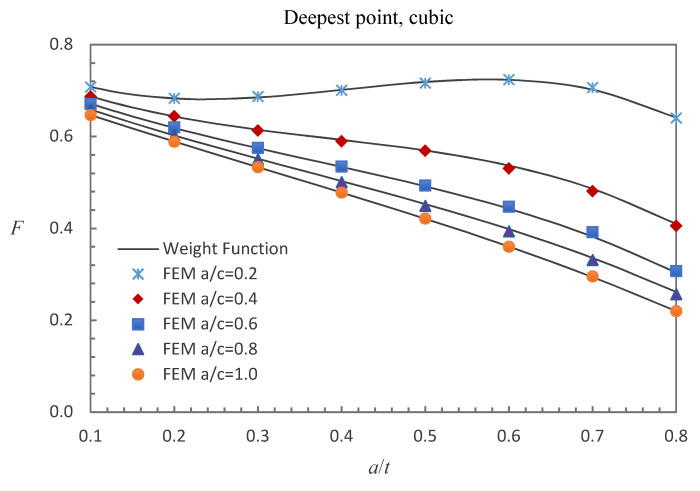
Comparison of weight function and FE results in this study with cubic stress distribution (deepest point).

**Figure 10:**
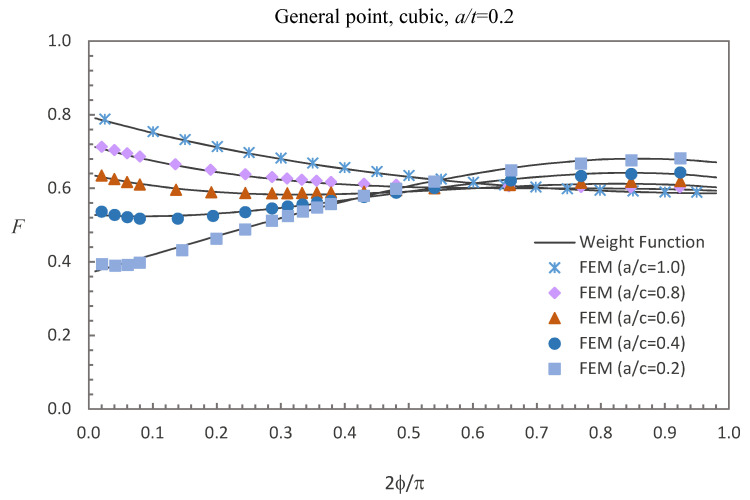
Comparison of weight function and FE results in this study with cubic stress distribution (general point).

An FGM with a Young’s modulus that is governed by the following equation was defined to validate the SIFs calculated from the derived weight functions.

(49) $$E\left(y\right)=E_{0}\exp\left[\left(\frac{1}{w}\right)\cdot \ln\left(\frac{E_{1}}{E_{0}}\right)y\right]$$

where $E_{0}$ = 210 GPa, $E_{1}$ = 200 GPa, and *w* = 100 mm. The accuracy of the derived weight functions is validated against the numerical results in this study. Comparisons between the FE results and the weight function results for the surface, deepest and general points affected by parabolic or cubic stress distribution are shown in Figure 5, Figure 6, Figure 7, Figure 8, Figure 9 and Figure 10, in which the comparisons of the BCFs for the general points are under the conditions of *a*/*t* = 0.2 and 0°<ϕ<90° (the range of parametric angle).

Figure 5, Figure 6 and Figure 7 show the comparisons between the FE results and the weight function results for the surface, deepest and general points affected by parabolic stress field. The difference between the BCFs calculated from the FE models and the BCFs calculated from the weight functions is generally less than 1.43% for the surface point, 2.46% for the deepest point and 3.52% for the general point.

Figure 8, Figure 9 and Figure 10 show the comparisons between the FE results and the weight function results for the surface, deepest and general points affected by cubic stress field. The difference between the BCFs obtained from the numerical simulations and the BCFs calculated from the weight functions is mostly less than 1.48% for the surface point, 2.61% for the deepest point and 3.70% for the general point.

It can be concluded that the results obtained from the weight functions are in reasonable agreement with the numerical solutions and the previously derived weight functions are accurate enough to predict the BCFs of cracked FG plates subjected to non-uniform stress distributions.

## 4. Conclusions

A weight function method has been developed in this study to calculate the SIFs of FG plates with semi-elliptical surface cracks. Constant, linear, parabolic and cubic stress fields have been applied to act on the crack face. The weight functions for the surface, deepest, and general points on a cracked FG plate have been derived and well validated. It can be concluded that the weight function method is accurate for evaluating the SIFs of cracked FG plates subjected to non-uniform stress distributions. The derived weight functions are applicable for fracture analyses of surface cracks on FGMs.

## Figures and Tables

**Table 1:** Parameters assigned in finite element (FE) models.

Parameter	Values
*a*/*c*	0.2, 0.4, 0.6, 0.8, 1.0
*a*/*t*	0.1, 0.2, 0.3, 0.4, 0.5, 0.6, 0.7, 0.8

**Table 2:** Comparison of boundary correction factors (BCFs) (*F*): current study vs. results $F_{s}$ in reference [11], for *a*/*c* = 0.2.

**(a) *F* of Current Study**					
σ(x)	Position	*a*/*t* = 0.2	*a*/*t* = 0.4	*a*/*t* = 0.6	*a*/*t* = 0.8
Linear	Surface	0.5011	0.5785	0.7083	0.9450
	Deepest	0.4724	0.6134	0.7956	0.8647
Parabolic	Surface	0.4553	0.5160	0.6153	0.7995
	Deepest	0.2956	0.4001	0.5381	0.5844
**(b) Difference Between *F* and $F_{s}$,** $\left|F-F_{s}\right|/F_{s}\times 100$					
Linear	Surface	1.9325	2.6073	2.3851	1.5147
	Deepest	1.6857	1.4302	1.4493	1.5597
Parabolic	Surface	1.5325	2.3673	2.1651	1.2147
	Deepest	2.1857	1.8607	1.9103	2.0397

## Supplementary Materials

Supplementary materials are available online at https://www.mdpi.com/1996-1944/13/14/3155/s1.
