# Supplementary material for: Weight Function Method for Stress Intensity Factors of Semi-Elliptical Surface Cracks on Functionally Graded Plates Subjected to Non-Uniform Stresses
Source: Materials (Basel). 2020 Jul 15;13(14):3155. doi: 10.3390/ma13143155 (PMC7411839; doi:10.3390/ma13143155)
Supplement: Supplementary file 1 [file materials-13-03155-s001.pdf]

materials

Supplementary Materials

Weight Function Method for Stress Intensity Factors of Semi-Elliptical Surface Cracks on Functionally Graded Plates Subjected to Non-Uniform Stresses

Kun-Pang Kou, Jin-Long Cao, Yang Yang and Chi-Chiu Lam

Nomenclature

a	crack depth of a semi-elliptical surface crack
c	half crack length of a semi-elliptical surface crack
D_1, D_2, D_3	weight function coefficients
D_{D1}, D_{D2}, D_{D3}	weight function coefficients for deepest point
D_{S1}, D_{S2}, D_{S3}	weight function coefficients for surface point
D_{P1}, D_{P2}, D_{P3}	weight function coefficients for general point
E	Young's modulus
E_0, E_1	Young's modulus of starting face constituent, ending face constituent
E_{tip}	Young's modulus at the crack tip
E'_{tip}	modified Young's modulus at the crack tip
F	boundary correction factors
F_0, F_1	boundary correction factors for reference stress intensity factors K_{r1}^{S} and K_{r2}^{S}
h	half height of functionally graded plate
K	stress intensity factor
$K_r(a)$	reference stress intensity factor related to crack length
K_{r1}^{D}, K_{r2}^{D}	reference stress intensity factors of deepest point
K_{r1}^{S}, K_{r2}^{S}	reference stress intensity factors of surface point
K_{r1}^{P}, K_{r2}^{P}	reference stress intensity factors of general point
Q	shape factor for an ellipse
t	thickness of functionally graded plate
w	half width of functionally graded plate
Y_0, Y_1	boundary correction factors for reference stress intensity factors K_{r1}^{D} and K_{r2}^{D}
Z_0, Z_1	boundary correction factors for reference stress intensity factors K_{r1}^{P} and K_{r2}^{P}
σ_0	nominal or characteristic stress
$\sigma(x)$	local stress distribution normal to the prospective crack face
υ	Poisson's ratio
ϕ	parametric angle of an elliptical surface crack

1. Detailed Derivation Process and Explanation

1.1. Detailed Explanation of Equation (15) in the Manuscript.

The relationship between the crack opening displacement $u(x,a)$ and the weight function $m(x,a)$ was derived by Rice [5], and it is expressed as follows:

$$m(x,a)=\frac{E_{\text{tip}}}{K_r(a)}\frac{\partial u(x,a)}{\partial a} \tag{S1}$$

Thus, the first derivative of the weight function with respect to x can be written in the following form.

$$\frac{\partial m(x,a)}{\partial x}=\frac{E_{\text{tip}}}{K_r(a)}\frac{\partial}{\partial a}\left[\frac{\partial u(x,a)}{\partial x}\right] \tag{S2}$$

The second derivative of the weight function with respect to x can be written in the following form.

$$\frac{\partial^2 m(x,a)}{\partial x^2}=\frac{E_{\text{tip}}}{K_r(a)}\frac{\partial}{\partial a}\left[\frac{\partial^2 u(x,a)}{\partial x^2}\right] \tag{S3}$$

The following derivation of the additional condition for a surface crack with depth a is from the reference [24]. Let us consider displacements u and v in the vicinity of the point (0, 0). Because the x-axis is an axis of symmetry, no shear stresses will be acting there.

$$\tau(x,0)=0 \tag{S4}$$

Along the free surface of the plate it holds:

$$\tau(0,y)=\sigma_x(0,y)=0 \tag{S5}$$

For stresses σ_x and τ developable by power series as:

$$\sigma_x=\sum_{v=0}^{\infty}\sum_{u=0}^{\infty}A_{uv}x^u y^v \qquad \tau=\sum_{v=0}^{\infty}\sum_{u=0}^{\infty}B_{uv}x^u y^v \tag{S6}$$

The following equation is obtained according to conditions (S4) and (S5):

$$B_{u0}=B_{0v}=A_{0v}=0 \tag{S7}$$

The relationship between deflections u, v and shear distortion γ is:

$$\frac{\partial u}{\partial x}+\frac{\partial v}{\partial y}=\gamma \tag{S8}$$

The following equation is obtained by taking the derivative of (S8) with respect to x and using the strain component $\varepsilon_x=\partial v/\partial x$.

$$\frac{\partial^2 u}{\partial x^2}=\frac{\partial\gamma}{\partial x}-\frac{\partial^2 v}{\partial x\partial y}=\frac{\partial\gamma}{\partial x}-\frac{\partial\varepsilon_x}{\partial y} \tag{S9}$$

By using Hooke's law, we obtain:

$$\varepsilon_x=m\sigma_x+n\sigma_y;\ \ \gamma=\tau/G \tag{S10}$$

$$m=\begin{cases}1/E\\(1-v^2)/E\end{cases};\ \ n=\begin{cases}-v/E & \text{for plane stress}\\-v(1+v)/E & \text{for plane strain}\end{cases}$$

The equilibrium condition gives:

$$\frac{\partial \sigma_y}{\partial y}+\frac{\partial \tau}{\partial x}=0 \tag{S11}$$

The following expression is obtained from Equations (S9), (S10) and (S11).

$$\frac{\partial^2 u}{\partial x^2}=\left(\frac{1}{G}+n\right)\frac{\partial \tau}{\partial x}-m\frac{\partial \sigma_x}{\partial y} \tag{S12}$$

The second derivative of u at point (0, 0) is as follows:

$$\frac{\partial^2 u}{\partial x^2}\bigg|_{x=y=0}=\left(\frac{1}{G}+n\right)B_{10}-mA_{01}=0 \tag{S13}$$

That is, directly at the surface of the plate, the curvature of the crack contour disappears [24].

Based on Equations (S5), (S6), (S7) and (S12), it can be proved that the curvature of the crack contour at the surface (x = 0) vanishes, as follows.

$$\frac{\partial^2 u(x,a)}{\partial x^2}\bigg|_{x=0}=0 \tag{S14}$$

Consequently, the second derivative of the weight function at x = 0 must also be zero. Thus, in the case of an edge or surface crack, Equation (S3) can be written as follows [6,24]:

$$\frac{\partial^2 m_{\mathrm{D}}(x,a)}{\partial x^2}\bigg|_{x=0}=0 \tag{S15}$$

1.2. Detailed Explanation of Equation (17) in the Manuscript.

The explanation of the sentence "Due to the weight function for the surface point of a semi-elliptical surface crack is derived from the weight function for the embedded penny-shape crack, therefore, the weight function in Equation (11) must vanish at $x=a$ [25]" in the manuscript is as follows.

The closed form weight function for an embedded circular crack (embedded penny-shape crack) is given [25]:

$$m_F(a,x,\theta)=\frac{1}{\pi\sqrt{\pi a}}\frac{\sqrt{(a^2-x^2)}}{a^2+x^2-2ax\cos\theta} \tag{S16}$$

Shen et al. [25] derived the weight function for the surface point B of a semi-elliptical surface crack from Equation (S16); it is expressed as follows.

$$m_{\mathrm{B}}(x,a)=\frac{2}{\sqrt{\pi x}}\left(1-\sqrt{\frac{x}{a}}\right)=\frac{2}{\sqrt{\pi x}}\left[1-\left(\frac{x}{a}\right)^{1/2}\right] \tag{S17}$$

The weight function for the surface point in the manuscript is given by analogy with the equation (21) in reference [25].

$$m_{\mathrm{S}}(x,a)=\frac{2}{\sqrt{\pi x}}\left(\frac{x}{a}\right)^{-1/2}\left[1+D_{\mathrm{S1}}\left(\frac{x}{a}\right)+D_{\mathrm{S2}}\left(\frac{x}{a}\right)^2+D_{\mathrm{S3}}\left(\frac{x}{a}\right)^3\right] \tag{S18}$$

Since the equation (21) in the reference must satisfy the condition that the weight function is zero at the crack tip (x = a) [25]; therefore, the weight functions in Equations (S17) and (S18) are equal to zero at $x=a$, leading to:

$$m_{\mathrm{S}}(x,a)\big|_{x=a}=0 \tag{S19}$$

1.3. Detailed Explanation of Equations (19) and (20) in the Manuscript.

The deepest point ($\phi = \pi/2$) and surface point ($\phi = 0$) are special cases of general points [26]. Fett et al. [24] pointed out that for a surface crack with depth *a*, the curvature of the crack contour at the surface ($x = 0$) vanishes. That is, the condition that the curvature of the crack contour at $x = 0$ is zero should be satisfied if the general point infinitely approaches the deepest point ($\phi \to \pi/2$).

$$\frac{\partial^2 u(x,a,\phi)}{\partial x^2}\Big|_{x=0,\phi\to\pi/2} = 0 \tag{S20}$$

Consequently, the second derivative of the weight function of the general point at $x = 0$ should also be zero.

$$\frac{\partial^2 m_{\mathrm{P1}}(x,a,\phi)}{\partial x^2}\Big|_{x=0,\phi\to\pi/2} = 0 \tag{S21}$$

Finally, we obtain:

$$\frac{\partial^2 m_{\mathrm{P1}}(x,a)}{\partial x^2}\Big|_{x=0} = 0 \tag{S22}$$

As explained in the previous derivation, the weight function for the surface point must be zero at the crack tip ($x = a$) [25]. The condition that the weight function of the surface point is zero at $x = a$ should be satisfied if the general point is infinitely approaching the surface point ($\phi \to 0$).

$$m_{\mathrm{P2}}(x,a,\phi)\Big|_{x=a,\phi\to 0} = 0 \tag{S23}$$

Consequently, the Equation (19) in the manuscript is obtained:

$$m_{\mathrm{P2}}(x,a)\Big|_{x=a} = 0 \tag{S24}$$

Notice: For references cited in supplementary materials, please refer to the corresponding references in the manuscript.

2. Detailed Derivation Process of Equations (41), (42), (43) and (44)

$$\frac{\partial}{\partial x^2}\left\{\sqrt{\frac{2}{\pi a\sin\phi}}\left[\left(1-\frac{x}{a\sin\phi}\right)^{-\frac{1}{2}} + D_{\mathrm{P1}}\left(1-\frac{x}{a\sin\phi}\right)^{\frac{1}{2}} + D_{\mathrm{P2}}\left(1-\frac{x}{a\sin\phi}\right)^{\frac{3}{2}}\right]\right\}\Bigg|_{x=0}$$

$$=\left\{\sqrt{\frac{2}{\pi a\sin\phi}}\frac{\partial}{\partial x}\left[-\frac{1}{2}\left(-\frac{1}{a\sin\phi}\right)\left(1-\frac{x}{a\sin\phi}\right)^{-\frac{3}{2}} + \frac{1}{2}D_{\mathrm{P1}}\left(-\frac{1}{a\sin\phi}\right)\left(1-\frac{x}{a\sin\phi}\right)^{-\frac{1}{2}}\right.\right.$$
$$\left.\left. + \frac{3}{2}D_{\mathrm{P2}}\left(-\frac{1}{a\sin\phi}\right)\left(1-\frac{x}{a\sin\phi}\right)^{\frac{1}{2}}\right]\right\}\Bigg|_{x=0}$$

$$=\left\{\sqrt{\frac{2}{\pi a\sin\phi}}\left[\frac{3}{4}\left(-\frac{1}{a\sin\phi}\right)^{2}\left(1-\frac{x}{a\sin\phi}\right)^{-\frac{5}{2}} - \frac{1}{4}D_{\mathrm{P1}}\left(-\frac{1}{a\sin\phi}\right)^{2}\left(1-\frac{x}{a\sin\phi}\right)^{-\frac{3}{2}}\right.\right.$$
$$\left.\left. + \frac{3}{4}D_{\mathrm{P2}}\left(-\frac{1}{a\sin\phi}\right)^{2}\left(1-\frac{x}{a\sin\phi}\right)^{-\frac{1}{2}}\right]\right\}\Bigg|_{x=0} = 0$$

$$\Rightarrow \qquad 3 - D_{\mathrm{P1}} + 3D_{\mathrm{P2}} = 0 \tag{41}$$

$$\left\{\sqrt{\frac{2}{\pi a\sin\phi}}\left[\left(\frac{x}{a\sin\phi}-1\right)^{-\frac{1}{2}}+D_{\mathrm{P3}}\left(\frac{x}{a\sin\phi}-1\right)^{\frac{1}{2}}+D_{\mathrm{P4}}\left(\frac{x}{a\sin\phi}-1\right)^{\frac{3}{2}}\right]\right\}\Bigg|_{x=a}$$

$$=\sqrt{\frac{2}{\pi a\sin\phi}}\left[\left(\frac{1}{\sin\phi}-1\right)^{-\frac{1}{2}}+D_{\mathrm{P3}}\left(\frac{1}{\sin\phi}-1\right)^{\frac{1}{2}}+D_{\mathrm{P4}}\left(\frac{1}{\sin\phi}-1\right)^{\frac{3}{2}}\right]=0$$

$$\Rightarrow\quad 1+D_{\mathrm{P3}}\left(\frac{1}{\sin\phi}-1\right)+D_{\mathrm{P4}}\left(\frac{1}{\sin\phi}-1\right)^{2}=0 \qquad (42)$$

$$K_{1r}^{\mathrm{P}}=\int_0^{a\sin\phi}\sigma_0\left\{\sqrt{\frac{2}{\pi a\sin\phi}}\left[\left(1-\frac{x}{a\sin\phi}\right)^{-\frac{1}{2}}+D_{\mathrm{P1}}\left(1-\frac{x}{a\sin\phi}\right)^{\frac{1}{2}}+D_{\mathrm{P2}}\left(1-\frac{x}{a\sin\phi}\right)^{\frac{3}{2}}\right]\right\}\mathrm{d}x$$

$$+\int_{a\sin\phi}^{a}\sigma_0\left\{\sqrt{\frac{2}{\pi a\sin\phi}}\left[\left(\frac{x}{a\sin\phi}-1\right)^{-\frac{1}{2}}+D_{\mathrm{P3}}\left(\frac{x}{a\sin\phi}-1\right)^{\frac{1}{2}}+D_{\mathrm{P4}}\left(\frac{x}{a\sin\phi}-1\right)^{\frac{3}{2}}\right]\right\}\mathrm{d}x$$

$$=\sigma_0\sqrt{\frac{2}{\pi a\sin\phi}}\left[-2a\sin\phi\left(1-\frac{x}{a\sin\phi}\right)^{\frac{1}{2}}-\frac{2}{3}a\sin\phi D_{\mathrm{P1}}\left(1-\frac{x}{a\sin\phi}\right)^{\frac{3}{2}}\right.$$

$$\left.-\frac{2}{5}a\sin\phi D_{\mathrm{P2}}\left(1-\frac{x}{a\sin\phi}\right)^{\frac{5}{2}}\right]\Bigg|_0^{a\sin\phi}$$

$$+\sigma_0\sqrt{\frac{2}{\pi a\sin\phi}}\left[2a\sin\phi\left(\frac{x}{a\sin\phi}-1\right)^{\frac{1}{2}}+\frac{2}{3}a\sin\phi D_{\mathrm{P3}}\left(\frac{x}{a\sin\phi}-1\right)^{\frac{3}{2}}\right.$$

$$\left.+\frac{2}{5}a\sin\phi D_{\mathrm{P4}}\left(\frac{x}{a\sin\phi}-1\right)^{\frac{5}{2}}\right]\Bigg|_{a\sin\phi}^{a}$$

$$=\sigma_0\sqrt{\frac{2}{\pi a\sin\phi}}\left[2a\sin\phi+\frac{2}{3}a\sin\phi D_{\mathrm{P1}}+\frac{2}{5}a\sin\phi D_{\mathrm{P2}}\right]$$

$$+\sigma_0\sqrt{\frac{2}{\pi a\sin\phi}}\left[2a\sin\phi\left(\frac{1}{\sin\phi}-1\right)^{\frac{1}{2}}+\frac{2}{3}a\sin\phi D_{\mathrm{P3}}\left(\frac{1}{\sin\phi}-1\right)^{\frac{3}{2}}\right.$$

$$\left.+\frac{2}{5}a\sin\phi D_{\mathrm{P4}}\left(\frac{1}{\sin\phi}-1\right)^{\frac{5}{2}}\right]=\sigma_0\sqrt{\frac{\pi a}{Q}}Z_0$$

$$\Rightarrow\left[1+\frac{1}{3}D_{\mathrm{P1}}+\frac{1}{5}D_{\mathrm{P2}}\right]+\left[\left(\frac{1}{\sin\phi}-1\right)^{\frac{1}{2}}+\frac{1}{3}D_{\mathrm{P3}}\left(\frac{1}{\sin\phi}-1\right)^{\frac{3}{2}}+\frac{1}{5}D_{\mathrm{P4}}\left(\frac{1}{\sin\phi}-1\right)^{\frac{5}{2}}\right]$$

$$=\pi\sqrt{\frac{1}{8Q\sin\phi}}Z_0=\sqrt{\frac{1}{Q\sin\phi}}V_0 \qquad (43)$$

$$K_{2r}^{\mathrm{P}}=\int_0^{a\sin\phi}\sigma_0\left(1-\frac{x}{a}\right)\left\{\sqrt{\frac{2}{\pi a\sin\phi}}\left[\left(1-\frac{x}{a\sin\phi}\right)^{-\frac{1}{2}}+D_{\mathrm{P1}}\left(1-\frac{x}{a\sin\phi}\right)^{\frac{1}{2}}+D_{\mathrm{P2}}\left(1-\frac{x}{a\sin\phi}\right)^{\frac{3}{2}}\right]\right\}\mathrm{d}x$$
$$+\int_{a\sin\phi}^{a}\sigma_0\left(1-\frac{x}{a}\right)\left\{\sqrt{\frac{2}{\pi a\sin\phi}}\left[\left(\frac{x}{a\sin\phi}-1\right)^{-\frac{1}{2}}+D_{\mathrm{P3}}\left(\frac{x}{a\sin\phi}-1\right)^{\frac{1}{2}}+D_{\mathrm{P4}}\left(\frac{x}{a\sin\phi}-1\right)^{\frac{3}{2}}\right]\right\}\mathrm{d}x$$

$$\int_0^{a\sin\phi}\sigma_0\left(1-\frac{x}{a}\right)\left\{\sqrt{\frac{2}{\pi a\sin\phi}}\left[\left(1-\frac{x}{a\sin\phi}\right)^{-\frac{1}{2}}+D_{\mathrm{P1}}\left(1-\frac{x}{a\sin\phi}\right)^{\frac{1}{2}}+D_{\mathrm{P2}}\left(1-\frac{x}{a\sin\phi}\right)^{\frac{3}{2}}\right]\right\}\mathrm{d}x$$
$$=\left\{\sigma_0\left(1-\frac{x}{a}\right)\sqrt{\frac{2}{\pi a\sin\phi}}\left[-2a\sin\phi\left(1-\frac{x}{a\sin\phi}\right)^{\frac{1}{2}}-\frac{2}{3}a\sin\phi D_{\mathrm{P1}}\left(1-\frac{x}{a\sin\phi}\right)^{\frac{3}{2}}\right.\right.$$
$$\left.\left.-\frac{2}{5}a\sin\phi D_{\mathrm{P2}}\left(1-\frac{x}{a\sin\phi}\right)^{\frac{5}{2}}\right]\right\}\Bigg|_0^{a\sin\phi}$$
$$+\frac{\sigma_0}{a}\int_0^{a\sin\phi}\left\{\sqrt{\frac{2}{\pi a\sin\phi}}\left[-2a\sin\phi\left(1-\frac{x}{a\sin\phi}\right)^{\frac{1}{2}}-\frac{2}{3}a\sin\phi D_{\mathrm{P1}}\left(1-\frac{x}{a\sin\phi}\right)^{\frac{3}{2}}\right.\right.$$
$$\left.\left.-\frac{2}{5}a\sin\phi D_{\mathrm{P2}}\left(1-\frac{x}{a\sin\phi}\right)^{\frac{5}{2}}\right]\right\}\mathrm{d}x$$
$$=\sigma_0\sqrt{\frac{2}{\pi a\sin\phi}}\left[2a\sin\phi+\frac{2}{3}a\sin\phi D_{\mathrm{P1}}+\frac{2}{5}a\sin\phi D_{\mathrm{P2}}\right]$$
$$-\frac{\sigma_0}{a}\sqrt{\frac{2}{\pi a\sin\phi}}\left[\frac{4}{3}a^2\sin^2\phi+\frac{4}{15}a^2\sin^2\phi D_{\mathrm{P1}}+\frac{4}{35}a^2\sin^2\phi D_{\mathrm{P2}}\right]$$
$$=a\sigma_0\sqrt{\frac{2}{\pi a\sin\phi}}\left[2\sin\phi+\frac{2}{3}\sin\phi D_{\mathrm{P1}}+\frac{2}{5}\sin\phi D_{\mathrm{P2}}\right]$$
$$-a\sigma_0\sqrt{\frac{2}{\pi a\sin\phi}}\left[\frac{4}{3}\sin^2\phi+\frac{4}{15}\sin^2\phi D_{\mathrm{P1}}+\frac{4}{35}\sin^2\phi D_{\mathrm{P2}}\right]$$

$$\int_{a\sin\phi}^{a}\sigma_0\left(1-\frac{x}{a}\right)\left\{\sqrt{\frac{2}{\pi a\sin\phi}}\left[\left(\frac{x}{a\sin\phi}-1\right)^{-\frac{1}{2}}+D_{\mathrm{P3}}\left(\frac{x}{a\sin\phi}-1\right)^{\frac{1}{2}}+D_{\mathrm{P4}}\left(\frac{x}{a\sin\phi}-1\right)^{\frac{3}{2}}\right]\right\}\mathrm{d}x$$

$$=\left\{\sigma_0\left(1-\frac{x}{a}\right)\sqrt{\frac{2}{\pi a\sin\phi}}\left[2a\sin\phi\left(\frac{x}{a\sin\phi}-1\right)^{\frac{1}{2}}+\frac{2}{3}a\sin\phi D_{\mathrm{P3}}\left(\frac{x}{a\sin\phi}-1\right)^{\frac{3}{2}}\right.\right.$$

$$\left.\left.+\frac{2}{5}a\sin\phi D_{\mathrm{P4}}\left(\frac{x}{a\sin\phi}-1\right)^{\frac{5}{2}}\right]\right\}\Bigg|_{a\sin\phi}^{a}$$

$$+\frac{\sigma_0}{a}\int_{a\sin\phi}^{a}\left\{\sqrt{\frac{2}{\pi a\sin\phi}}\left[2a\sin\phi\left(\frac{x}{a\sin\phi}-1\right)^{\frac{1}{2}}+\frac{2}{3}a\sin\phi D_{\mathrm{P3}}\left(\frac{x}{a\sin\phi}-1\right)^{\frac{3}{2}}\right.\right.$$

$$\left.\left.+\frac{2}{5}a\sin\phi D_{\mathrm{P4}}\left(\frac{x}{a\sin\phi}-1\right)^{\frac{5}{2}}\right]\right\}\mathrm{d}x$$

$$=a\sigma_0\sqrt{\frac{2}{\pi a\sin\phi}}\left[\frac{4}{3}\sin^2\phi\left(\frac{x}{a\sin\phi}-1\right)^{\frac{3}{2}}+\frac{4}{15}\sin^2\phi D_{\mathrm{P3}}\left(\frac{x}{a\sin\phi}-1\right)^{\frac{5}{2}}\right.$$

$$\left.+\frac{4}{35}\sin^2\phi D_{\mathrm{P4}}\left(\frac{x}{a\sin\phi}-1\right)^{\frac{7}{2}}\right]\Bigg|_{a\sin\phi}^{a}$$

$$\Rightarrow\left[1+\frac{1}{3}D_{\mathrm{P1}}+\frac{1}{5}D_{\mathrm{P2}}\right]-\left[\frac{2}{3}\sin\phi+\frac{2}{15}\sin\phi D_{\mathrm{P1}}+\frac{2}{35}\sin\phi D_{\mathrm{P2}}\right]$$

$$+\left[\frac{2}{3}\sin\phi\left(\frac{1}{\sin\phi}-1\right)^{\frac{3}{2}}+\frac{2}{15}\sin\phi D_{\mathrm{P3}}\left(\frac{1}{\sin\phi}-1\right)^{\frac{5}{2}}+\frac{2}{35}\sin\phi D_{\mathrm{P4}}\left(\frac{1}{\sin\phi}-1\right)^{\frac{7}{2}}\right]$$

$$=\pi\sqrt{\frac{1}{8Q\sin\phi}}Z_1=\sqrt{\frac{1}{Q\sin\phi}}V_1 \qquad (44)$$

© 2020 by the authors. Submitted for possible open access publication under the terms and conditions of the Creative Commons Attribution (CC BY) license (http://creativecommons.org/licenses/by/4.0/).
